# High-Quality Draft Genome Sequences for Five Non-O157 Shiga Toxin-Producing *Escherichia coli* Strains Generated with PacBio Sequencing and Optical Maps

**DOI:** 10.1128/genomeA.00626-16

**Published:** 2016-06-30

**Authors:** Rebecca L. Lindsey, Lori Rowe, Lisley Garcia-Toledo, Vladimir Loparev, Kristen Knipe, Devon Stripling, Haley Martin, Eija Trees, Phalasy Juieng, Dhwani Batra, Nancy Strockbine

**Affiliations:** a Centers for Disease Control and Prevention, Atlanta, Georgia, USA; b Oak Ridge Institute for Science and Education, Oak Ridge, Tennessee, USA

## Abstract

Shiga toxin-producing *Escherichia coli* (STEC) is a foodborne pathogen. We report here the high-quality draft whole-genome sequences of five STEC strains isolated from clinical cases in the United States. This report is for STEC of serotypes O55:H7, O79:H7, O91:H14, O153:H2, and O156:H25.

## GENOME ANNOUNCEMENT

The overall incidence rate of Shiga toxin-producing *Escherichia coli* (STEC) infection in the United States in 2011 was 1.8 per 100,000 people. Symptoms of STEC infection range from mild diarrhea to severe bloody diarrhea, which in some individuals can progress to hemolytic-uremic syndrome. More than 100 STEC serotypes are known to cause illness in humans, but most infections are caused by seven serotypes (1, 2). We report here the availability of five high-quality draft whole-genome sequence assemblies generated by PacBio sequencing and verified using the strain optical map. The sequenced strains include serogroups O91 and O153, which in 2011 were the 11th and 15th, respectively, most common STEC serotypes isolated from human infections in the United States (http://www.cdc.gov/ncezid/dfwed/pdfs/national-stec-surveillance-overiew-508c.pdf).

STEC genomic DNA was extracted according to the manufacturer’s protocol (ArchivePure; 5 Prime, Gaithersburg, MD). DNAs were sheared to 10 kbp utilizing g-Tubes (Covaris, Inc., Woburn, MA). The 10-kbp sheared products were further size selected utilizing 0.45× AMPure beads. Sheared DNAs were used to generate large SMRTbell libraries using the standard library protocols of the Pacific Biosciences DNA template preparation kit (Menlo Park, CA). An average of 3 single-molecule real-time (SMRT) cells were used to sequence each isolate. Finished libraries were bound to proprietary P5 polymerase and sequenced on a PacBio RSII sequencer using C3 chemistry for 150-min movies. Sequence reads were filtered and assembled *de novo* utilizing the PacBio Hierarchical Genome Assembly Process version 3 (3). The sequence order in the resulting assemblies was verified using restriction enzymes AflII and NcoI whole-genome maps (WGM). WGM were generated according to the OpGen protocol. 

The accession numbers and assembly metrics for each draft genome sequence are listed in Table 1. Genomic overlap was found in the single contig for isolates 2013C-4465 and 2011C-3911, but the chromosomes were not closed. The plasmids associated with these isolates as well as those of 2011C-3198 are closed. The PacBio sequence order did not exactly match the optical maps for isolates 2011C-4315 (insertion) and 2014C-3250 (inversion), but all base pairs are included. STEC sequences were annotated with the NCBI Prokaryotic Genome Automatic Annotation Pipeline (4).

**TABLE 1:** Accession numbers and assembly metrics of the five annotated STEC draft whole-genome sequences

*E. coli* isolate	Serotype	NCBI accession no.	No. of contigs	Genome size (bp)	*N*_50_	G+C content (%)	Associated plasmid size(s) (bp)
2013C-4465	O55:H7	CP015241, CP015242	1	5,494,965	5,428,937	50.45	66,029
2011C-3911	O79:H7	CP015239, CP015240	1	4,989,575	4,863,599	50.88	125,977
2011C-3198	O91:H14	LXPF00000000	3	5,292,261	3,592,774	50.74	131,906
2011C-4315	O153:H2	LXPG00000000	2	5,543,270	5,052,897	50.89	86,415, 113,463
2014C-3250	O156:H25	LXPH00000000	2	5,351,793	3,690,314	50.71	90,729

A detailed report on additional analysis of the draft genome sequences will be included in a future publication.

### Nucleotide sequence accession numbers.

The annotated whole-genome *E. coli* sequences have been deposited in DDBJ/ENA/GenBank under the accession numbers listed in Table 1. The version described in this paper is the first version.
